# Identifying subgroups of patients with type 2 diabetes based on real-world traditional chinese medicine electronic medical records

**DOI:** 10.3389/fphar.2023.1210667

**Published:** 2023-06-29

**Authors:** Shuai Zhao, Hengfei Li, Xuan Jing, Xuebin Zhang, Ronghua Li, Yinghao Li, Chenguang Liu, Jie Chen, Guoxia Li, Wenfei Zheng, Qian Li, Xue Wang, Letian Wang, Yuanyuan Sun, Yunsheng Xu, Shihua Wang

**Affiliations:** ^1^ Department of Endocrinology, Second Affiliated Hospital of Shandong University of Traditional Chinese Medicine, Jinan, China; ^2^ Department of Infectious Diseases, Hubei Provincial Hospital of Traditional Chinese Medicine (Affiliated Hospital of Hubei University of Chinese Medicine, Hubei Province Academy of Traditional Chinese Medicine), Wuhan, China; ^3^ Hebei Provincial Hospital of Traditional Chinese Medicine, Shijiazhuang, China; ^4^ Institute of Basic Research in Clinical Medicine, China Academy of Chinese Medical Sciences, Beijing, China; ^5^ Institute of Traditional Chinese Medicine, Shandong University of Traditional Chinese Medicine, Jinan, China; ^6^ Department of Nursing, Second Affiliated Hospital of Shandong University of Traditional Chinese Medicine, Jinan, China; ^7^ Department of Obstetrics and Gynecology, Weifang Fangzi District People’s Hospital, Weifang, China

**Keywords:** type 2 dabetes, real-world clinical data, heterogeneous medical record network method, traditional chinese medcine, enrichment analysis

## Abstract

**Introduction:** Type 2 diabetes (T2D) is a multifactorial complex chronic disease with a high prevalence worldwide, and Type 2 diabetes patients with different comorbidities often present multiple phenotypes in the clinic. Thus, there is a pressing need to improve understanding of the complexity of the clinical Type 2 diabetes population to help identify more accurate disease subtypes for personalized treatment.

**Methods:** Here, utilizing the traditional Chinese medicine (TCM) clinical electronic medical records (EMRs) of 2137 Type 2 diabetes inpatients, we followed a heterogeneous medical record network (HEMnet) framework to construct heterogeneous medical record networks by integrating the clinical features from the electronic medical records, molecular interaction networks and domain knowledge.

**Results:** Of the 2137 Type 2 diabetes patients, 1347 were male (63.03%), and 790 were female (36.97%). Using the HEMnet method, we obtained eight non-overlapping patient subgroups. For example, in H3, Poria, Astragali Radix, Glycyrrhizae Radix et Rhizoma, Cinnamomi Ramulus, and Liriopes Radix were identified as significant botanical drugs. Cardiovascular diseases (CVDs) were found to be significant comorbidities. Furthermore, enrichment analysis showed that there were six overlapping pathways and eight overlapping Gene Ontology terms among the herbs, comorbidities, and Type 2 diabetes in H3.

**Discussion:** Our results demonstrate that identification of the Type 2 diabetes subgroup based on the HEMnet method can provide important guidance for the clinical use of herbal prescriptions and that this method can be used for other complex diseases.

## 1 Introduction

Type 2 diabetes (T2D) is the most common type of diabetes and accounts for approximately 90% of all diabetes cases worldwide; T2D is a complex, serious and multifactorial chronic disease that has become an increasingly prevalent health issue and imposes a tremendous economic burden worldwide (Li et al., 2015; International Diabetes Federation IDF, 2019). People with T2D have an approximately 15% higher overall excess mortality risk than people who do not have T2D (Tancredi et al., 2015). Although T2D is defined by a single metabolite, glucose, it is increasingly recognized as a highly heterogeneous disease with varying clinical manifestations (Gregg et al., 2014; World Health Organization, 2019a; Ahlqvist et al., 2021). Therefore, identifying the precise subtypes of T2D patients would be important for preventing serious complications, predicting individualized drug responses and improving health outcomes for patients with diabetes in the early stage and help predict the drug responses of patients with diabetes (Pigeyre et al., 2022; Williams et al., 2022).

Precision medicine has been recognized as a new medical approach for refining the disease taxonomy and improving the healthcare capability (National Research Council US, 2011; Zhou et al., 2018). Recently, several studies have identified new subtypes of T2D through data-driven analysis of a clinical population, which has improved the understanding of T2D with the goal of improving patient care in clinical settings (Li et al., 2015; Ahlqvist et al., 2018). These studies suggested that there are opportunities to further refine the current definition of T2D in real-world clinical settings into additional subtypes (American Diabetes Association, 2010). Traditional Chinese medicine (TCM) is a typical kind of personalized medicine (Jiang et al., 2012; Zhou et al., 2014) that classifies disease conditions into different subtypes (i.e., syndromes) through the comprehensive analysis of symptom phenotypes identified by the four main diagnostic TCM procedures (observation, listening, questioning, and pulse analyses). Furthermore, individualized treatment (in most cases, with herbal prescriptions) would be ordered for patients according to the diagnosis of syndromes. This clinical framework presents a novel view of disease conditions from symptom profiles and herbal prescriptions for patients.

In this study, we collected large-scale real-world TCM clinical data on T2D and used an established heterogeneous medical record network (HEMnet) (Edward et al., 2017) method to identify the clinical subgroups of T2D. Four types of clinical features, namely, symptom phenotypes, syndrome diagnoses, herbal prescriptions and comorbid disease conditions, together with phenotype–genotype associations and botanical drug -efficacy relationships, were incorporated into the HEMnet approach to help identify clinical groups with both clinical meaningfulness and biological insights. Enrichment analysis was used to identify the significant features of the clinical characteristics and molecular pathways of the T2D patient groups. Our findings are expected to help refine the understanding of T2D by both improving personalized treatment and identifying the underlying mechanisms.

## 2 Materials and methods

### 2.1 Clinical data and preprocessing

The data of 2137 inpatients diagnosed with T2D were collected from the EMR database of the Second Affiliated Hospital of Shandong University of TCM from 2016 to 2021, which included all inpatient information obtained during hospitalization, such as demographic information, symptoms, laboratory or physical tests, diagnoses and treatment. Because most data were in free text that cannot be used directly for analysis, we used a clinical information extraction tool (Shu et al., 2019) to efficiently extract the biomedical entities (e.g., symptoms, diseases) from these records. Then, to normalize the various clinical term descriptions, we manually checked and standardized the terms “disease”, “botanical drug” and “drug” by referring to the 10th Revision of International Classification of Diseases (ICD-10) (World Health Organization, 2019b), the Pharmacopoeia of the People’s Republic of China 2020 Revision (ChP 2020) (Chinese Pharmacopoeia Commission, 2020), and DrugBank Online (Wishart et al., 2018), respectively. In addition, diseases with detailed ICD-10 codes were further aggregated into higher level codes. For example, the ICD-10 codes I50.903 and I50.905 were aggregated into ICD-10 code I50.9.

### 2.2 External data sources

In this study, several external data sources were used to support this research. The efficacy of botanical drugs was extracted from ChP 2020, and human protein‒protein interactions (PPIs) were obtained from the STRING database (Szklarczyk et al., 2019). The phenotype–genotype and botanical drug–target associations were extracted from the SymMap database (Wu et al., 2019). The disease–gene associations were extracted from the MalaCards database (Rappaport et al., 2017).

### 2.3 The HEMnet method

**FIGURE 1:**
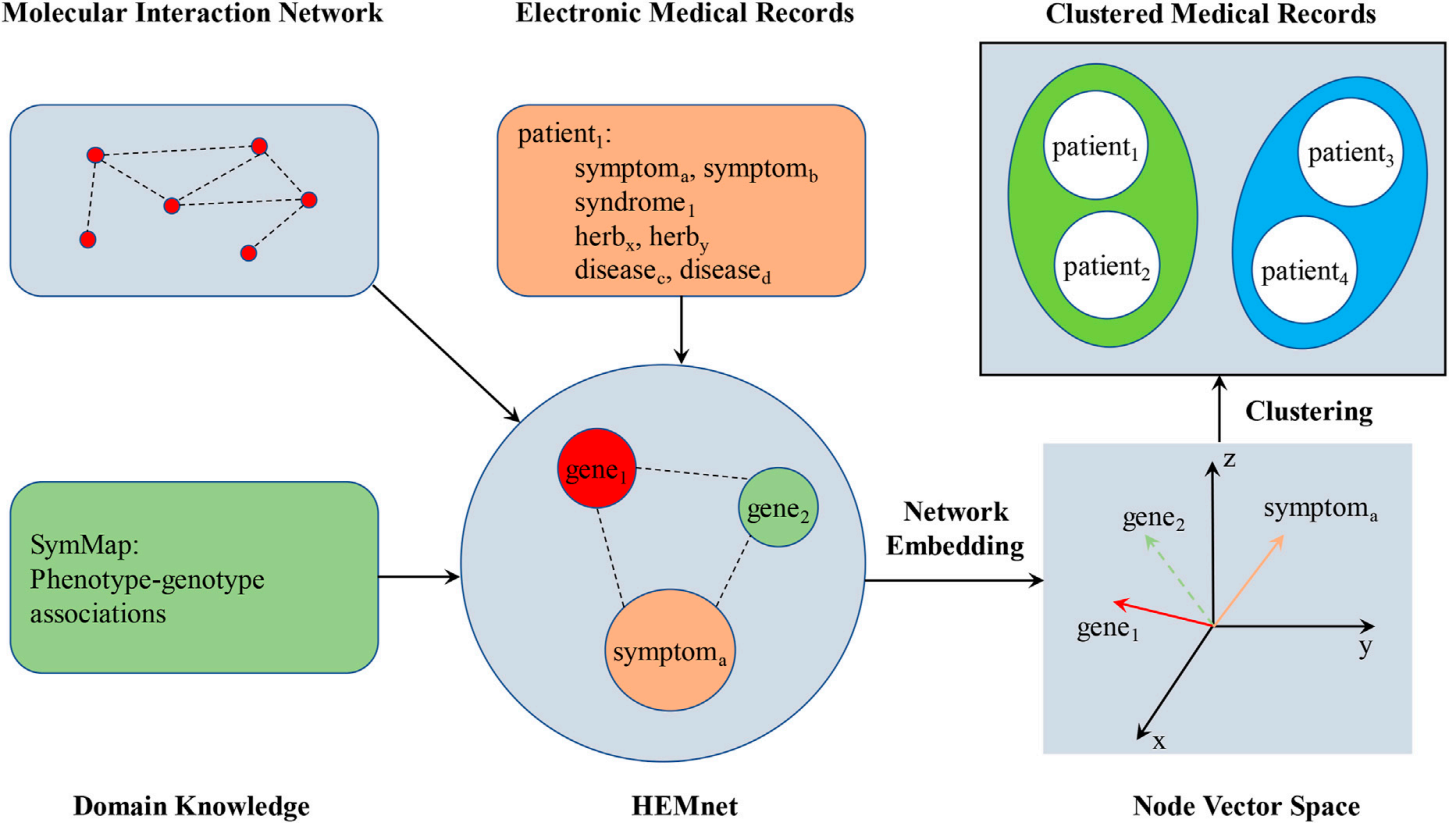
The pipeline for the HEMnet.

Missing data and semantic mismatch were the two main challenges of EMR analysis. Therefore, we used HEMnet to address the challenges of EMR analysis by leveraging information from several external sources to supplement clinical data (Edward et al., 2017). In our study, we utilized three distinct categories of edges to create the HEMnet (Figure 1). The first two categories PPI and phenotype–genotype were drawn from the external database, while the last category was drawn directly from the EMRs.

1) PPI. This network was based on HumanNet, an external network of protein-encoding genes (Lee et al., 2011). The nodes are proteins, and the undirected edges are the interactions between proteins.
2) Phenotype–genotype associations. This network was obtained from SymMap. The nodes were phenotype or genotype, and the undirected edges were the association of the phenotype and genotype.
3) Co-occurrence of clinical entities from the EMR. We directly added the clinical cooccurrence edges of botanical drugs from each medical record. The missing data was one of the main challenges of electronic medical records (EMR) analysis, especially the lack of symptom information. Botanical drugs can represent symptom precision to address missing symptom information in EMR. We repeated this for all clinical features in each patient’s medical record.

Then, HEMnet uses an embedding method, ProSNet (Wang et al., 2017), to infer relationships among its constituent nodes. ProSNet takes a heterogeneous network as input, on which it performs a novel dimensionality reduction algorithm to optimize a low-dimensional vector representation for each node. The vectors of two nodes are colocalized in the low-dimensional space if the nodes are close to each other in the heterogeneous network. After generating low-dimensional vector representations of nodes in the HEMnet, a similarity matrix was constructed according to the similarity between every two embedding vector features, which was calculated by cosine similarity. Finally, the similarity matrix was used to fill in missing features of the original patient characteristics and form the phenotypes of patients (Edward et al., 2017).

The K-means clustering (MacQueen, 1967) was used for the patient phenotype. According to the outcomes, the patients were divided into eight non-overlapping subgroups. The t-distributed stochastic neighbour embedding (t-SNE) algorithm (Cieslak et al., 2020) was used to visualize the outcomes.

The chi-square test and relative risk (RR) (Pirhaji et al., 2008; Ouimet et al., 2010) were used to assess the significance of clinical features, including symptom phenotypes, syndrome diagnoses, botanical drugs and comorbidities in eight subgroups. In this study, patients with a certain clinical feature, such as a symptom phenotype, in a particular subgroup as an exposed group, and the remaining patients with this certain clinical feature as the non-exposed group. So RR is defined as *RR = (C*_*ij*_ */C*_*i*_ *)/((C*_*j*_ *-C*_*ij*_ *)/(N-C*_*i*_ *))*, where *C*_*i*_ is the number of patients in subgroup *i*, *C*_*j*_ is the number of patients with a clinical feature *j*, *C*_*ij*_ represents the number of patients in subgroup *i* and with a clinical feature *j* and *N* is the total number of patients in the study. A *p*-value <0.05, which was obtained from the chi-square test, and an RR > 1 indicated that a clinical feature was truly significant.

### 2.4 Gene ontology (GO) and KEGG pathway enrichment analysis

The GO and KEGG pathway enrichment analysis are useful to trackle the DNA-related and protein-related problems. And they offers considerable power for discovering the biological functions of genes and proteins (Chen et al., 2017). The Gene Ontology (GO) project serves as a comprehensive source for functional genomics. The project creates evidence-supported annotations to describe the biological roles of individual genome products (e.g., genes, proteins, ncRNAs, complexes) (Gene Ontology Consortium, 2015). The KEGG pathway database is the main database in Kyoto Encyclopedia of Genes and Genomes (KEGG), and it consists of manually drawn reference pathway maps together with organism-specific pathway maps (Kanehisa et al., 2017). We obtained enriched GO and KEGG pathways using the Database for Annotation, Visualization, and Integrated Discovery (DAVID), which is a web-based online bioinformatics resource that aims to provide tools for the functional interpretation of large lists of genes/proteins (Sherman et al., 2022).

## 3 Results

### 3.1 Basic characteristics

As shown in the table below (Table 1), of the 2137 T2D patients, 1347 (63.03%) were male, and 790 (36.97%) were female. The ages of most T2D patients (60.60%) were between 60 and 79 years old. The average length of stay (LOS) was 14.08 ± 9.20, and for most patients (41.83%), LOS was between 8 and 14 days. We counted the distinct number of comorbidities of each patient and found that most patients had 6–10 diagnoses (56.43%).

**TABLE 1:** The characteristics of the 2137 T2D inpatients.

Characteristics		n (%)/(mean ± SD)
Sex	Male	1347 (63.03)
	Female	790 (36.97)
Age		66.31 ± 11.44
Age group	<20	1 (0.05)
	20–39	30 (1.40)
	40–59	527 (24.66)
	60–79	1295 (60.60)
	≥80	284 (13.29)
LOS		14.08 ± 9.20
LOS group	1–7	495 (23.16)
	8–14	894 (41.83)
	15–21	391 (18.30)
	22–28	186 (8.70)
	≥29	171 (8.00)
Number of comorbidities	1–5	764 (35.75)
	6–10	1206 (56.43)
	≥11	167 (7.81)

Then, we analysed the distribution of the top five clinical features including symptom phenotypes, syndrome diagnoses, botanical drugs, and comorbidities (Table 2).

**TABLE 2:** The top five clinical features.

Clinical features		n (%)
Symptom phenotypes	Insomnia	763 (35.70)
	Poor absorbing	487 (22.79)
	Lack of energy	416 (19.47)
	Chest tightness	239 (11.18)
	Constipation	215 (10.06)
Syndrome diagnoses	Deficient qi and blood stasis	618 (28.92)
	Qi-Yin deficiency	247 (11.56)
	Qi stagnation and blood stasis	97 (4.54)
	Blood stasis	77 (3.60)
	Wind and phlegm blocked channel	43 (2.01)
Botanical drug	Poria	1294 (60.55)
	Astragali radix	1133 (53.02)
	Angelicae sinensis radix	1073 (50.21)
	Glycyrrhizae radix et rhizoma	969 (45.34)
	Glycyrrhizae radix et rhizoma praeparata cum melle	848 (39.68)
Comorbidities	Essential (primary) hypertension	1569 (73.42)
	Atherosclerotic heart disease	1127 (52.74)
	Cerebral infarction	743 (34.75)
	Heart failure	664 (31.07)
	Unstable angina	429 (20.07)

### 3.2 The result of the HEMnet

With the method introduced in the Materials and Methods, we utilized three distinct categories of edges to create the HEMnet, which contained 5,846 nodes and 125,426 connected edges. There were 3,000 symptom nodes and 2,846 gene nodes. Furthermore, there were 16,641 PPI edges, 8,749 phenotype–genotype edges, and 100,036 symptom edges.

**FIGURE 2:**
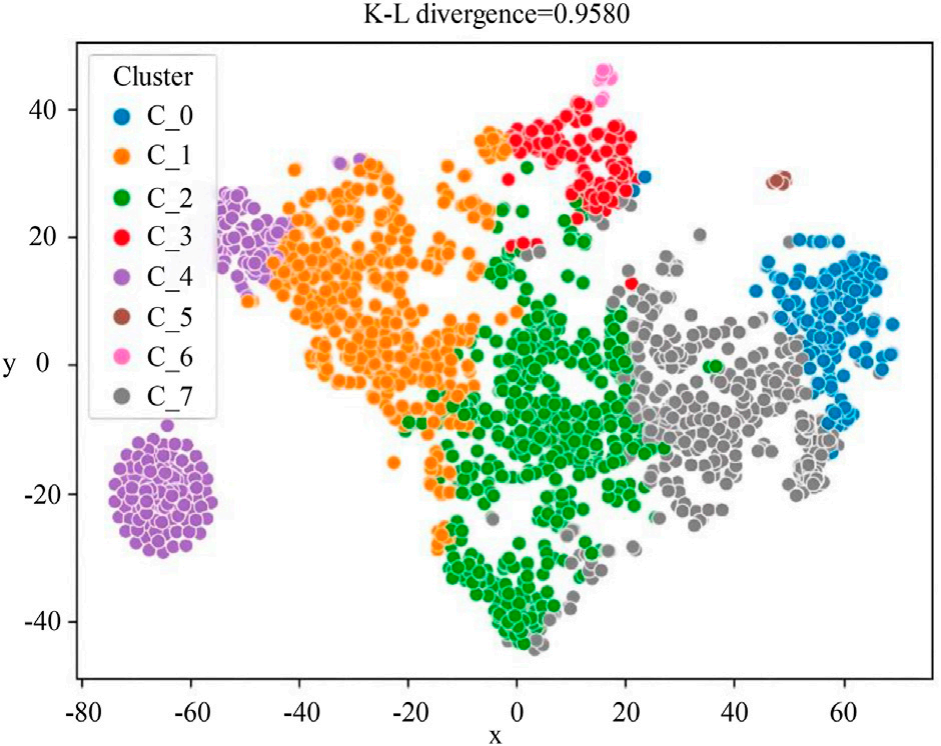
The visualized clustering result of HEMnet. The correspondence between the C_0-C_7 clusters in the figure and the H1-H8 subgroups in this paper is as follows: C_0 = H5, C_1 = H2, C_2 = H1, C_3 = H6, C_4 = H4, C_5 = H8, C_6 = H7, C_7 = H3. This picture was to reduce the dimensionality of the patient’s characterization vector to a two-dimensional vector for display. So the *x*-axis and *y*-axis represent the patient’s characterization vector, and the closer the two points are, the closer the patient’s characteristics are.

Then, the embedding method ProSNet was used to generate low-dimensional vector representations of nodes in the HEMnet. A similarity matrix was constructed according to the similarity between every two embedding vector features, which was calculated by cosine similarity, and used to fill in missing features of the original patient characteristics to form the patient phenotypes. Finally, using the K-means clustering algorithm, eight non-overlapping patient subgroups were obtained. The t-SNE algorithm was used to visualize the clustering results (Figure 2). The numbers of patients in the eight subgroups were as follows (Table 3): H1 (n = 547, 25.60%), H2 (n = 501, 23.44%), H3 (n = 432, 20.22%), H4 (n = 298, 13.94%), H5 (n = 197, 9.22%), H6 (n = 132, 6.18%), H7 (n = 18, 0.84%), and H8 (n = 12, 0.56%).

**TABLE 3:** The numbers of patients in the eight subgroups.

Subgroups	n (%)
H1	547 (25.60)
H2	501 (23.44)
H3	432 (20.22)
H4	298 (13.94)
H5	197 (9.22)
H6	132 (6.18)
H7	18 (0.84)
H8	12 (0.56)

### 3.3 The significant clinical features of the subgroups

We then selected the top 10 clinical features in these modules according to their frequency in each subgroup. Then, the RR and chi-square test (RR > 1 and *p* < 0.05, see Materials and methods) were used to screen the significant clinical features.

Because of fewer patients in H7 and H8 subgroups, it was less meaningful to analyse them. And since this study focused on the precision treatment of comorbidities, the H1, H2, and H4 subgroups with no significant botanical drugs and the H5 subgroup with a lower frequency of botanical drug use were excluded according to the screening results. Finally, H3 and H6 were included for further analysis.

We present the statistically significant botanical drugs, comorbidities, syndromes, and symptoms in H3 and H6 (Table 4, Table 5, Table 6, and Table 7), Poria, Astragali Radix, Glycyrrhizae Radix et Rhizoma, Cinnamomi Ramulus, and Ophiopogonis radix were the significant botanical drugs. Essential (primary) hypertension, atherosclerotic heart disease, heart failure, unstable angina, etc., were the significant comorbidities. Qi-Yin deficiency was the main significant syndrome. And chest tightness, fever, coarse lung breathing, vomiting, expectoration, etc., were the significant symptoms. In H6, Chuanxiong Rhizoma, Gastrodiae Rhizoma, and Baked Ziziphi Spinosae Semen were the significant botanical drugs. Cerebral infarction, sequelae of cerebral infarction and sequelae of intracerebral haemorrhage were the significant comorbidities. Deficient qi and blood stasis was the main significant syndrome. And poor physical activity, fever, slurring of speech, vomiting, etc., were the significant symptoms.

**TABLE 4:** The significant botanical drugs in H3 and H6.

Subgroup	Botanical drug	n (%)	*p*	RR
H3	Poria	150 (34.72)	4.35E-03	1.25
	Astragali Radix	131 (30.32)	1.36E-02	1.24
	Glycyrrhizae Radix et Rhizoma	118 (27.31)	5.74E-03	1.29
	Cinnamomi Ramulus	100 (23.15)	1.50E-02	1.29
	Ophiopogonis radix	89 (20.60)	2.72E-02	1.28
H6	Chuanxiong Rhizoma	35 (26.52)	2.84E-02	1.41
	Gastrodiae Rhizoma	28 (21.21)	1.01E-09	3.17
	Baked Ziziphi Spinosae Semen	24 (18.18)	1.62E-03	1.89

**TABLE 5:** The significant comorbidities in H3 and H6.

Subgroup	Comorbidity	n (%)	*p*	RR
H3	Essential (primary) Hypertension	344 (79.63)	1.07E-03	1.11
	Atherosclerotic Heart Disease	265 (61.34)	6.05E-05	1.21
	Heart Failure	194 (44.91)	3.48E-12	1.63
	Unstable Angina	127 (29.40)	6.09E-08	1.66
	Cardiac Arrhythmia	65 (15.05)	3.24E-04	1.64
	Atrial Fibrillation and Flutter	52 (12.04)	6.80E-03	1.52
H6	Cerebral Infarction	93 (70.45)	6.21E-19	2.17
	Sequelae of Cerebral Infarction	23 (17.42)	3.00E-08	3.21
	Sequelae of Intracerebral Haemorrhage	17 (12.88)	4.60E-25	15.19

**TABLE 6:** The significance syndromes in H3 and H6.

Subgroup	Syndrome	n (%)	*p*	RR
H3	Qi-Yin deficiency	63 (14.58)	2.77E-02	1.35
	Qi-blood deficiency	9 (2.08)	2.02E-02	2.96
	Defideficiency of spleen and kidney	9 (2.08)	4.75E-05	8.88
	Wind-cold attacking lung	7 (1.62)	7.31E-03	4.60
	Phlegm-damp obstructing lung	6 (1.39)	1.37E-02	4.74
	Phlegm-heat obstructing lung	6 (1.39)	6.04E-03	5.92
H6	Deficient qi and blood stasis	70 (53.03)	3.44E-10	1.93
	Wind and phlegm bloke channel	13 (9.85)	2.99E-10	6.58
	Phlegm and blood stasis blocking collaterals	8 (6.06)	6.89E-05	4.86
	Blood stasis blocking collaterals	7 (5.30)	1.58E-03	3.94
	Deficiency of liver and kidney	5 (3.79)	6.18E-06	10.85
	Stirring wind due to yin deficiency	4 (3.03)	1.41E-06	20.25
	Kidney deficiency	2 (1.52)	1.60E-03	30.38

**TABLE 7:** The significant symptoms in H3 and H6.

Subgroup	Symptom	n (%)	*p*	RR
H3	Chest tightness	315 (72.92)	4.56E-33	1.79
	Fever	268 (62.04)	7.32E-10	1.36
	Coarse lung breathing	253 (58.56)	6.36E-25	1.85
	Vomiting	250 (57.87)	2.01E-16	1.60
	Expectoration	242 (56.02)	5.11E-25	1.90
	Dizziness	231 (53.47)	1.50E-09	1.43
	Fatigue	225 (52.08)	5.34E-11	1.49
	Insomnia	224 (51.85)	1.42E-23	1.94
	Cough	218 (50.46)	9.21E-22	1.90
	Headache	156 (36.11)	6.33E-08	1.55
H6	Poor physical activity	103 (78.03)	1.86E-164	14.90
	Fever	78 (59.09)	1.47E-02	1.23
	Slurring of speech	76 (57.57)	3.18E-80	8.55
	Vomitting	69 (52.27)	4.53E-03	1.32
	Poor activity	66 (50.00)	8.53E-66	8.08
	Fatigue	64 (48.48)	1.36E-02	1.29
	Coarse lung breathing	63 (47.73)	9.46E-03	1.31
	Disability of left limbs	55 (41.67)	2.35E-104	21.98
	Choking cough	50 (37.88)	9.95E-36	5.75

### 3.4 Significant GO terms and pathways for H3 and H6

In this part, we explored the shared molecular associations between the significant botanical drugs and comorbidities of T2D in H3 and H6. First, we identified the distinct genes associated with each significant botanical drug and comorbidity in H3 and H6 from an external database (see Materials and methods). Then, we obtained the pathways and GO terms for the botanical drugs, comorbidities and T2D in H3 and H6 by the DAVID program (2021, see Materials and methods). Finally, we screened out pathways and GO terms with *p* < 0.05 from botanical drugs, comorbidities and T2D. We identified the overlapping pathways and GO terms among the botanical drugs, comorbidities, and T2D in H3 and H6 (Table 8 and Table 9). In H3, there were six overlapping pathways and eight overlapping GO terms among the botanical drugs, comorbidities, and T2D. In H6, there were no overlapping pathways among the botanical drugs, comorbidities, and T2D. Therefore, we reported on the pathways that overlapped between the two of them. There was only one overlapping GO term among the botanical drugs, comorbidities, and T2D. For example, most of the pathways and GO functions in H3 were associated with T2D, such as type II diabetes mellitus, insulin resistance, glucose metabolic process, and response to glucose. The significant botanical drugs in H3 had some overlapping pathways and GO terms with comorbidities and T2D.

**TABLE 8:** The overlapping pathways among the botanical drugs, comorbidities, and T2D in H3 and H6.

Subgroup	Pathway	Botanical drug	Comorbidity	T2D
H3	cGMP-PKG signalling pathway	1.26E-02	4.25E-13	2.71E-02
	Diabetic cardiomyopathy	3.24E-05	2.27E-03	7.76E-03
	Insulin resistance	1.82E-07	2.30E-03	1.94E-10
	MicroRNAs in cancer	6.13E-03	2.21E-03	1.59E-04
	Regulation of lipolysis in adipocytes	3.43E-04	3.96E-05	1.32E-03
	Type II diabetes mellitus	1.02E-04	1.13E-04	6.42E-14
H6	Adipocytokine signalling pathway	4.96E-02	ns	2.41E-03
	Diabetic cardiomyopathy	ns	6.53E-03	7.76E-03
	FoxO signalling pathway	2.23E-02	ns	1.41E-04

Ns: not significant.

**TABLE 9:** The overlapping GO terms among the botanical drugs, comorbidities, and T2D in H3 and H6.

Subgroup	GO	Botanical drug	Comorbidity	T2D	Category
H3	glucose metabolic process	1.03E-08	3.24E-03	1.49E-05	BP
	liver development	1.31E-03	1.93E-04	1.13E-03	BP
	negative regulation of gene expression	3.93E-07	3.31E-09	5.93E-04	BP
	positive regulation of cell proliferation	1.51E-15	4.49E-03	1.30E-03	BP
	positive regulation of gene expression	5.73E-16	1.68E-09	9.17E-04	BP
	response to drug	2.72E-33	7.92E-05	4.48E-05	BP
	response to glucose	5.33E-07	5.09E-03	1.68E-08	BP
	response to xenobiotic stimulus	2.51E-30	7.37E-05	2.01E-03	BP
H6	response to xenobiotic stimulus	9.85E-04	3.15E-02	2.01E-03	BP

## 4 Discussion

In recent years, the continual growth of EMR databases has facilitated clinical research, paved the way for data mining applications, and supported population health. However, missing data is the biggest barrier to using EMRs (Kruse et al., 2018). In our study, the problem of missing data and semantic mismatch in EMRs posed a considerable challenge. For example, if T2D was not the primary diagnosis, the patient’s T2D-related symptoms would not be recorded in the medical record, which results in incomplete information in the patient’s medical record. Furthermore, the overabundant expression of symptoms, diagnoses, botanical drugs, and syndromes in clinical TCM data leads to mismatched records containing semantically similar but lexically distinct terms. Therefore, the problem of missing data and semantic mismatch were solved by standardizing the data and creating the HEMnet to ensure the reliability of the research results (Edward et al., 2017).

Analysing disease comorbidities with EMR data has become popular in real-world clinical settings for chronic disease conditions such as T2D and chronic liver diseases (Li et al., 2015; Ahlqvist et al., 2018; Shu et al., 2019; Mansour Aly et al., 2021). In this manuscript, the HEMnet method was used to identify the eight non-overlapping patient subgroups. Then, H3 and H6 were screened according to a specific screening strategy for subgroups to further analyse the clinical features. For example, cardiovascular disease (CVD), such as atherosclerotic heart disease, heart failure, unstable angina, cardiac arrhythmia, atrial fibrillation and flutter, was a significant comorbidity of T2D in H3. In large prospective trials, T2D has been identified as a significant risk factor for CVD, including stroke, angina, heart failure, myocardial infarction, and atherosclerosis (Emerging Risk Factors Collaboration Sarwar et al., 2010; Peters et al., 2014; Shah et al., 2015; Einarson et al., 2018). Regarding treatment, Poria, Astragali radix, Glycyrrhizae radix et rhizoma, Cinnamomi ramulus, and Ophiopogonis radix were the significant botanical drugs in H3. And studies have shown that these botanical drugs used alone or in combination with other botanical drugs are often used to treat diabetes as well as other disorders (Jia et al., 2003; Li et al., 2004; Lindequist et al., 2005).

Furthermore, to explore the shared molecular associations among the significant botanical drugs, comorbidities and T2D in H3 and H6, we explored the overlapping pathways and GO terms between the significant botanical drugs and comorbidities of T2D in H3 and H6. The significant botanical drugs in H3 had six pathways and eight GO terms that overlapped between comorbidities and T2D. This result indicated that these botanical drugs may have therapeutic effects on comorbidities and T2D via the pathways and GO terms identified in the analysis. For example, the overlapping pathways in H3 inculded insulin resistance which is one shared defect in T2D and Essential (primary) Hypertension. Although the mechanisms by which defective insulin action *per se* contributes to high blood pressure are still somewhat uncertain (Ferrannini and Cushman, 2012). But previous studies have demonstrated that within the physiological concentration range of insulin, it causes slight increases in limb blood flow by enhancing the release of nitric oxide (via stimulation of nitric oxide synthase activity in endothelial cells) and by potentiating acetylcholine-induced vasodilation. In people with insulin resistance, vasodilation in response to supraphysiological insulin concentrations is reduced (Taddei et al., 1995; Yki-Järvinen and Utriainen, 1998; Steinberg and Baron, 2002; Giacco and Brownlee, 2010). Astragaloside Ⅳ (AST Ⅳ, chemical formula: C41H68O14, molecular weight:785), as the primary active ingredient of Astragali radix, has the pharmacological effects of regulating lipid and carbohydrate metabolism and improving insulin resistance. Previous studies have shown that AST Ⅳ improvement of insulin resistance may be related to activation of the IRS1/protein kinase B (AKT) insulin signaling pathway to increase the glucose transporter type 4 (GLUT4) activity, thus increasing glucose uptake and insulin sensitivity (Zhou et al., 2021). So the main findings of the GO and KEGG pathway enrichment analysis require further experimental verification.

Our study has several potential limitations. Our sample included only 2137 hospitalized patients, resulting in an insufficient number of patients with some subtypes of T2D for identification of additional significant TCM phenotypes. In future studies, more patients should be included to ensure the abundance of the results. Another limitation is that Western medicine and laboratory tests were not included in our study. Therefore, the resulting disease subtypes would incorporate little information on these features. In addition, some patients were not given herbal prescriptions. This might affect the results of data mining. Finally, we used EMRs from only one hospital, and the resulting patient subgroups that were identified may not be representative. And further experiments should be performed to verify the results of this paper (Sheng et al., 2021).

## 5 Conclusion

Our results demonstrate that Cardiovascular disease (CVD) and Qi-Yin deficiency syndrome were significant comorbidity and TCM syndrome of T2D in subgroup H3, respectively. Regarding treatment, Poria, Astragali radix, Glycyrrhizae radix et rhizoma, Cinnamomi ramulus, and Ophiopogonis radix were the significant botanical drugs in subgroup H3. In subgroup H6, cerebral infarction and its sequelae, Qi deficiency and blood stasis syndrome were significant comorbidities and TCM syndrome, respectively. Regarding treatment, Chuanxiong rhizoma, Gastrodiae rhizoma, and Baked ziziphi spinosae semen were the significant botanical drugs. So identification of the T2D subgroup based on the HEMnet method can provide important guidance for the clinical use of herbal prescriptions and that this method can be used for other complex diseases.

## Data Availability

The raw dataset obtained from the electronic medical record of the hospital presented in this article is not available because of local legislation and institutional requirements. Requests to access the datasets should be directed to the corresponding author. The external datasets, such as human protein-protein interactions, phenotype-genotype, and botanical drug-target associations supporting the conclusions of this article will be made available by the authors, without undue reservation.
